# BigQ: a NoSQL based framework to handle genomic variants in i2b2

**DOI:** 10.1186/s12859-015-0861-0

**Published:** 2015-12-29

**Authors:** Matteo Gabetta, Ivan Limongelli, Ettore Rizzo, Alberto Riva, Daniele Segagni, Riccardo Bellazzi

**Affiliations:** Dipartimento di Ingegneria Industriale e dell’Informazione and Center for Health Technologies, Università di Pavia, Pavia, Italy; Dipartimento di Medicina Molecolare, Università di Pavia, Pavia, Italy; IRCCS Fondazione S. Maugeri, Pavia, Italy; IRCCS Fondazione Policlinico S. Matteo, Pavia, Italy; Interdisciplinary Center for Biotechnology Research, University of Florida, Gainesville, FL USA; Biomeris s.r.l., Pavia, Italy

**Keywords:** i2b2, NGS, Variants, CouchDB, NoSQL

## Abstract

**Background:**

Precision medicine requires the tight integration of clinical and molecular data. To this end, it is mandatory to define proper technological solutions able to manage the overwhelming amount of high throughput genomic data needed to test associations between genomic signatures and human phenotypes. The i2b2 Center (Informatics for Integrating Biology and the Bedside) has developed a widely internationally adopted framework to use existing clinical data for discovery research that can help the definition of precision medicine interventions when coupled with genetic data. i2b2 can be significantly advanced by designing efficient management solutions of Next Generation Sequencing data.

**Results:**

We developed BigQ, an extension of the i2b2 framework, which integrates patient clinical phenotypes with genomic variant profiles generated by Next Generation Sequencing. A visual programming i2b2 plugin allows retrieving variants belonging to the patients in a cohort by applying filters on genomic variant annotations. We report an evaluation of the query performance of our system on more than 11 million variants, showing that the implemented solution scales linearly in terms of query time and disk space with the number of variants.

**Conclusions:**

In this paper we describe a new i2b2 web service composed of an efficient and scalable document-based database that manages annotations of genomic variants and of a visual programming plug-in designed to dynamically perform queries on clinical and genetic data. The system therefore allows managing the fast growing volume of genomic variants and can be used to integrate heterogeneous genomic annotations.

**Electronic supplementary material:**

The online version of this article (doi:10.1186/s12859-015-0861-0) contains supplementary material, which is available to authorized users.

## Background

Precision medicine requires to tightly couple phenotypic and genotypic patient data [1], thus advocating for the development of IT tools that enable deep joint investigations of the two data sources. The Informatics for Integrating Biology and Beside (i2b2) system provides an excellent framework to analyze clinical data for research purposes [2, 3] and facilitates the implementation of precision medicine strategies [4]. Thanks to its modular architecture based on open-source REST web services and on the design of a simple but effective data warehouse scheme, i2b2 is now very popular in academia and industry, and has been used as the basis for many research projects, including for example TRANSMART [5].

Built on a “hive” of multiple server-side software modules (“cells”) that communicate through their integrated XML-based web services, the i2b2 platform consists of several core and optional cells. Each cell either holds data or a business tier. For example, the i2b2 web client interface allows performing *ad hoc* queries in order to find those patients having particular phenotypes described by an integrated controlled vocabulary. Once a patient set has been defined, data can be passed to one of the i2b2 plug-ins that implements specific analysis methods.

An interesting extension of the i2b2 capabilities is the ability to efficiently handle, together with clinical information, large-scale molecular data, and in particular those produced by Next Generation Sequencing (NGS) technologies. NGS technologies, able to read billions of DNA fragments at once, cover a broad range of genomic, transcriptomic and epigenomic applications allowing the study of genetic signals underlying phenotypic traits of interests. Over the last few years, targeted re-sequencing has become one of the most popular NGS genomic approaches due to its cost affordability [6]. In brief, it consists of selectively sequencing genomic regions of interest (e.g. genes), mapping the resulting DNA sequences to a given genomic reference, and reporting the identified differences, i.e. variants. The most exhaustive and common targeted re-sequencing application is whole-exome, that allows identifying variants over the entire set of known human genes [7].

The increasing availability of NGS facilities and the upcoming use of target re-sequencing technologies in clinical practice will generate large data sets that need to be properly integrated in software architectures able to jointly manage phenotypic and genotypic data for precision medicine purposes. On the one hand, it is important to report the presence of variants that have an established clinical meaning in the patients’ clinical records. On the other hand, it is also crucial to progressively store the variants with unknown meaning for future use and interpretation. A single whole-exome analysis may generate tens of thousands of such variants, which may need to be queried and retrieved, for example, within a large-scale study. Storing and retrieving this kind of data present a number of challenges. First, variants need to be annotated using genomic knowledgebases necessary for their interpretation. Second, since biomedical knowledge is steadily increasing, the data model used for variant representation should be flexible enough to support frequent updates and the introduction of new sources of biological annotations. Finally, variant queries need to be fast and the overall data management process should scale efficiently due to the growing number of experiments conducted.

Several approaches and frameworks have been developed with the aim to store, retrieve and analyze genomic variants [8–13]. Among them we can distinguish those based on relational databases [9, 11, 12] and Not-Only-SQL (NoSQL) ones [8, 13].

NoSQL solutions, in particular, represent a group of very interesting tools to store and retrieve very large data sets [14] and have emerged in recent years due to the rising need to handle “big data”, characterized by properties such high volume, variability and velocity [15].

Genomic variants can be rightfully included in this category. Volume and velocity are given by the high rate at which variants are generated by the increasingly fast and high throughput sequencing instruments. Variability refers to the need to pre-process and evaluate variants accordingly to different variant types (exon, splicing, intergenic variants etc.), sequencing applications, and diseases under study. Indeed, one may wish to use different genomic knowledgebases (e.g. COSMIC database for cancer related variants [16] and OMIM annotations in case of inherited diseases [17]), or to evaluate specific variant measures (e.g. allele frequencies in a control sample). Furthermore, because several genomic annotations fit only with a particular set of variant types, this would lead to many missing data in a structured data context (i.e. sparseness).

Even though efforts to standardize the way to report genomic variant and related NGS measures have been pursued [18, 19], variant annotation for genomic knowledgebases depends on the specific application and is difficult to standardize. As a consequence, structured and centralized relational databases are not the best choice to deal with increasingly growing, heterogeneously annotated genomic variants.

The flexible and distributed data model behind NoSQL databases, on the contrary, is suitable to systematically store and retrieve genomic variants and their annotations coming from different and frequently updated NGS analysis workflows. However, there is a tradeoff between data model flexibility and query complexity: NoSQL databases generally do not have a SQL-like query language, and queries have to be pre-computed in order to build the corresponding in-memory index structures that allow for fast searches.

Notably, NoSQL databases have been used to manage genomic data [8, 20], showing better performance than relational databases both in terms of horizontal scalability (i.e. adding more database instances) and computational time in data retrieval.

In their works, O’Connor B. et al. [8] and Wang S. et al. [20] adopted HBase, a *column-family* NoSQL database. Briefly, the underlying key-value data model [21] allows retrieving values (columns) that belong to rows by querying the related row-keys. Typically, one has to build a number of column families equal to the number of the desired queries. This leads to writing a significant amount of code to manage the population of column families and to ensure data consistency among them, both in import and update phase.

CouchDB [22] is a NoSQL database that uses semi-structured documents (JSON files) to handle data, a RESTful programming interface and JavaScript to define queries that exploit the MapReduce paradigm. It has been successfully adopted to deal with gene annotations, drug-target interactions, and copy number variants [23].

Here we present BigQ, an extension to the i2b2 framework, implemented to handle genomic variants such as single nucleotide variants (SNV) and short insertion and deletions (indel) within their annotations. BigQ allows the joint query of phenotype and genotype data by integrating different technological layers including for the first time a NoSQL component into the i2b2 overall architecture.

We chose CouchDB among the other NoSQL databases for several reasons. First, the object-oriented nature of JSON documents looks particularly appropriate to manage different kinds of genomic variants and their annotations. Second, the flexible schema of CouchDB allows storing information on variants, that is potentially heterogeneous, as JSON files. Third, queries are designed by writing simple JavaScript functions using MapReduce operations on JSON attributes, with the corresponding results indexed for a fast data retrieval. Notably, CouchDB automatically updates its indexes when JSON documents are added, deleted or modified. In particular, some features of CouchDB made it preferable among the other document-based NoSQL data stores, such as MongoDB: the embedded MapReduce engine, its REST-ful architecture and natively version-aware document management.

The CouchDB REST-ful programming interface has been used to enable communication between the variant database and the i2b2 framework. We extended i2b2 with two main components: i) a new i2b2 cell that manages HTTP requests to CouchDB and ii) a plug-in based on a visual programming paradigm that allows dynamically performing queries on clinical and genomic data. The i2b2 extension is also provided with an Extraction, Transformation and Loading (ETL) middleware that, starting from raw variant files in a standard format, uploads genomic variants, including a pre-defined set of annotations, in CouchDB. In the following we describe the technical aspects of this extension, and an evaluation of its performance on exome data from the 1000 Genomes Projects (1KGP) [24].

## Implementation

**Fig. 1:**
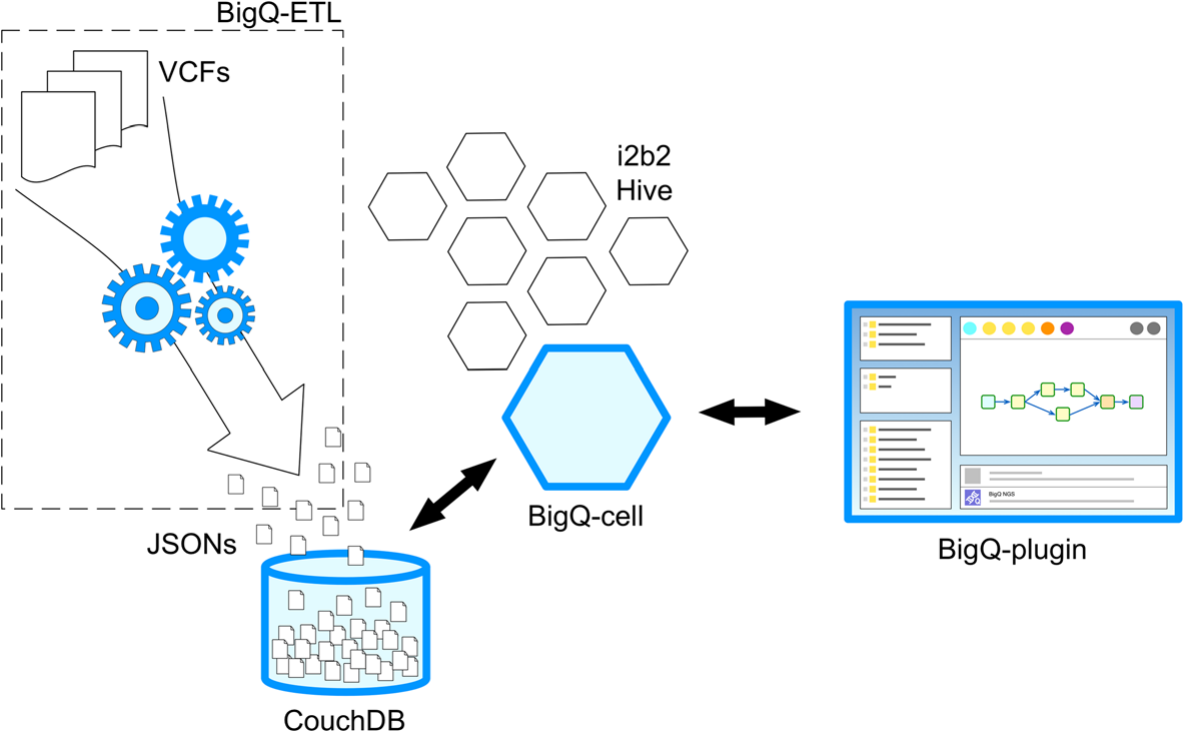
System components and their interrelationships. BigQ-ETL requires the user to provide one or more VCF files that are functionally annotated with ANNOVAR and used to create one JSON document for each variant belonging to each patient; these JSONs are stored in CouchDB to be queried by the BiqQ-Cell. On the client-side, the BigQ-plugin allows the user to create a genetic query with drag-and-drop interactions within the i2b2 Webclient; the plugin then communicates with the cell to run the query and collect the results that are shown to the user

BigQ consists of three main components: BigQ-ETL, to annotate and import variants in CouchDB; BigQ-cell, that holds the business logic to query CouchDB; and BigQ-plugin to query genomic data belonging to a patient cohort retrieved from the i2b2 data warehouse. Figure 1 shows the system components and their inter-relationships.

### The BigQ-ETL

Accordingly to the i2b2 policy, data entry is not demanded to the final user; therefore this module has been developed as a back-end tool.

BigQ-ETL takes genomic variants in the Variant Calling Format (VCF) [18] as input, and uses ANNOVAR [25] to annotate them. ANNOVAR allows to easily annotating a set of variants using several sources of information on transcripts, genes, gene-based functions (e.g. non-synonymous, nonsense etc.), evolutionary conservation scores, public variant databases such as dbSNP [26] and many other -omics resources [27, 28].

In particular, BigQ-ETL implements the *table_annovar* script, able to annotate variants both on standard and customized genomic tracks.

The data import process only requires providing the VCF files and some basic information, such as the i2b2 identification codes of the patients whose variants are contained in the VCF files. Once these data are available, the process is completely automatic: the files are sent to the server entrusted with the functional annotation process, where the *table_annovar* script is executed. Variants are enriched with transcripts, gene-based functions, 1KGP variant frequencies and the whole dbNSFP [28] dataset including variant scores given by several prediction tools such as PolyPhen-2 [29] and SIFT [30].

**Fig. 2:**
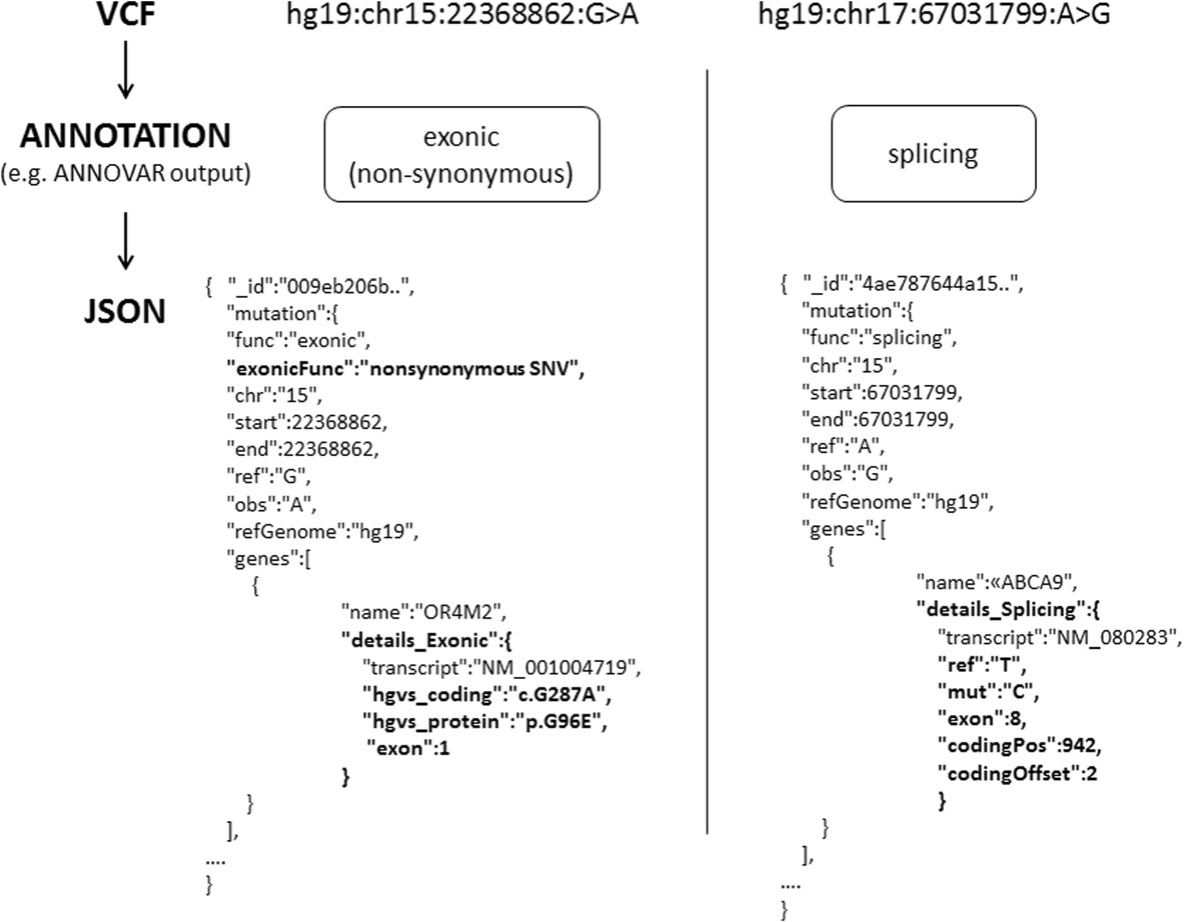
Example of annotated variants in JSON format. Two variants, represented in VCF format, are identified by standard attributes (reference genome, chromosome, variant position and nucleotide changes). The annotation step finds out that one variant falls in an exon causing an amino acid substitution at the protein level (non-synonymous) while the other is located in a transcript splicing site. The two variants generate two different JSON objects, characterized by different attributes. Differences between JSONs are highlighted in bold

The output of ANNOVAR is then used to create one JSON document for each variant belonging to a single patient. JSON is an open standard format used to transmit data objects consisting of attribute-value pairs. In our case, the set of possible attributes consist of data coming from the original VCF file, and of the functional annotations added by ANNOVAR (see Additional file 1: Table S1). Finally, each document is associated with a unique universal identifier (UUID) and sent to CouchDB. Notably, different types of variants may be represented by different data structures. For example, a deep intergenic variant may not have any gene associated with it, or a synonymous coding variant does not hold data about prediction scores such as PolyPhen-2 and SIFT (suitable only for non-synonymous variants). We have therefore developed an extensible object-oriented data structure, written in Java, able to model different kind of variants and annotations. One may want to add a new genomic track, or may not be interested in using others. Therefore, each variant type is modeled and treated individually, and holds only the needed attributes (see Fig. 2).

Alongside with the JSON documents, BigQ-ETL generates a set of *design documents* and sends them to CouchDB. These documents describe the queries (called *views*) that can be executed on the database.

Views are defined using JavaScript functions that specify attribute-value constraints corresponding to the query requirements, and that implement the *map* and *reduce* functions according to the MapReduce paradigm. *Map* functions are called once on each document: the document can be skipped (if it does not respect the constrain) or can be transformed (*emit*) into one or more view rows as key/value pairs. View rows are inserted into a B-tree storage engine and sorted by key (indexing): lookups by key or key range are therefore extremely efficient, with O(log N) complexity. Since *map* functions are applied to each document in isolation, computation can be highly parallelized within and across nodes where the database is distributed. Reduce functions can be optionally used in combination with *map* functions in order to report data aggregates grouping by row keys, such as counting the number of rows within a view or to calculate averages on related values.

We have chosen to specify a design document, and consequently an index, for all the possible attributes describing variants (see Additional file 1: Table S1). This choice allows us to perform any complex query as the combination of simple ones. It has to be noted that CouchDB flexibility allows building a view an all JSON documents despite their heterogeneity.

The whole data import process, which is potentially time and resource consuming, has been designed to be easily run in parallel, by splitting VCF files in batches, on a cloud-based architecture.

### Interval queries in BigQ

A genomic interval identifies a variant by its start and end positions with respect to the reference genome. Genome browsers [31, 32] and genomic annotation tools [25, 33, 34] are based on a particular binning scheme [35, 36] in order to index genomic intervals and allow for a fast search of overlapping features given a query interval.

We have implemented a similar approach in CouchDB that fits with its query logic.

**Fig. 3:**
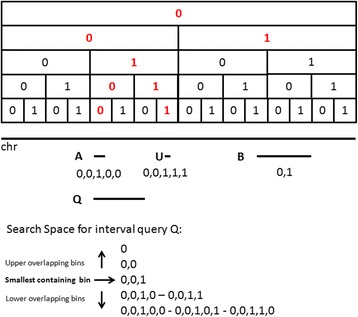
The simplified binning scheme and search strategy implemented in CouchDB. For the genomic feature A, the smallest containing bin is the one reached by navigating the tree in the following way: 0,0,1,0,0 (in red). For Feature B and Feature U the smallest bins are (0,1) and (0,0,1,1,1) respectively. Given the interval query Q, its smallest containing bin is the one coded by (0,0,1). When searching for genomic features within the corresponding overlapping bins, both for the lower and upper part of the tree, genomic feature U would also be reported: in fact, despite overlapping with one of the searched bins, it does not overlap with Q. Therefore, two more queries (views) are performed in order to remove the non-overlapping elements: the first adds the start position of the genomic feature to the view keys (*patient id, chromosome, bin, start*) while the second one adds the stop position. In this example, genomic feature U would be removed from the query result set because its start position is greater than the end one of Q

Each chromosome has been divided into a predefined set of hierarchical bins (called “tree”) depending on the specific chromosome length. The value 0 or 1 has been assigned to each bin, depending on being the left or right child bin within the tree, respectively (with the exception of the root bin, encoded by 0). A code is then assigned to each genomic feature (i.e. variants), corresponding to the ordered series of 0s and 1s given by navigating the binning tree from the root to the smallest bin that entirely contains the variant (see Fig. 3). The bin code is then represented as a JSON attribute, whose value is an array of 0s and 1s.

We implemented a CouchDB view with row keys composed by *patient id, chromosome* and *bin code*. Given an interval query, the search space is calculated a priori. It consists of the smallest containing bin for the interval query and its overlapping bins both for the upper and lower part of the binning tree. The view is therefore searched for the variants within these bins and two additional views are used to filter out variants that, even if located in the overlapping bins, do not overlap the interval query (see Fig. 3).

### The BigQ-cell

One of the most significant features of i2b2 is its loosely coupled structure, in which a set of web services (called *cells*) concurs to create the i2b2 server side core (called *hive*). The schema of exposed data is defined by the i2b2 XML-based messaging standard. This type of architecture lends itself easily to be updated and enhanced with new features [37, 38]. The BigQ-cell is a novel i2b2 cell we developed that enables the communication with CouchDB in order to execute queries on genetic data. The cell has been implemented in Java and uses the LightCouch library [39] to manage the communication with the database.

The cell extracts all the parameters required to execute a query from the XML file that it takes as its input:

- The basic object exchanged is a set of variants’ UUIDs grouped by patient, called *dataIn*.
- The *logic* of the query: “add” or “filter”. If “add” is chosen, the UUIDs returned by CouchDB are added to *dataIn* and sent back in the cell response; if “filter” is chosen, only the UUIDs belonging to both sets are sent back.
- The *query type* that identifies the variant fields (see Additional file 1: Table S1) on which the query should be executed. Examples of allowed query types are: *gene* for gene names, *exonicFunc* for exonic functions and *PolyPhenScore* for the PolyPhen-2 score.
- The *query details*, the set of values required to perform the specific type of query. For example: the list of gene names for the *gene* query, the list of exonic functions for the *exonicFunc* query and the endpoints of the score interval for the *PolyPhenScore* query.

Once these parameters are extracted, the cell accesses the CouchDB view associated with the specific *query type* according to the *query details*; this operation is performed for each patient in the *dataIn* set. The aggregated results from the database, consisting of a new set of variants’ UUIDs grouped by patient, is combined with *dataIn* according to the query *logic* to build the output object of the cell, called *dataOut*. Finally, the BigQ-cell builds the response XML message encoding the *dataOut* object and sends it back to the client.

### The BigQ-plugin

The BigQ plugin for the i2b2 Webclient has been specifically developed to communicate with the BigQ-cell. This plugin allows users to run genetic queries within i2b2, exploiting the patient sets previously extracted with phenotype queries. i2b2.

The main feature of BigQ-plugin, when compared to other Webclient plugins, is that its interface is based on visual programming. The user graphically builds queries with drag-and-drop interactions; feedback and results are presented within the same workspace in order to provide a more consistent experience. The plugin interface exploits the mxGraph Javascript libraries [40].

Each query exposed by the BigQ-cell is represented by a block that can be dragged into the workspace; the final query is made up by the sequence different blocks connected to each other. Besides standard blocks (which run queries in the cell), BigQ-plugin also provides the *Patient Result Set Drop* (PRS Drop) block, an input block to import a patient set in the workspace, and the *Patient Result Set Table* (PRS Table) block, an output block that shows the patients that have at least one variant that matches the query.

**Fig. 4:**
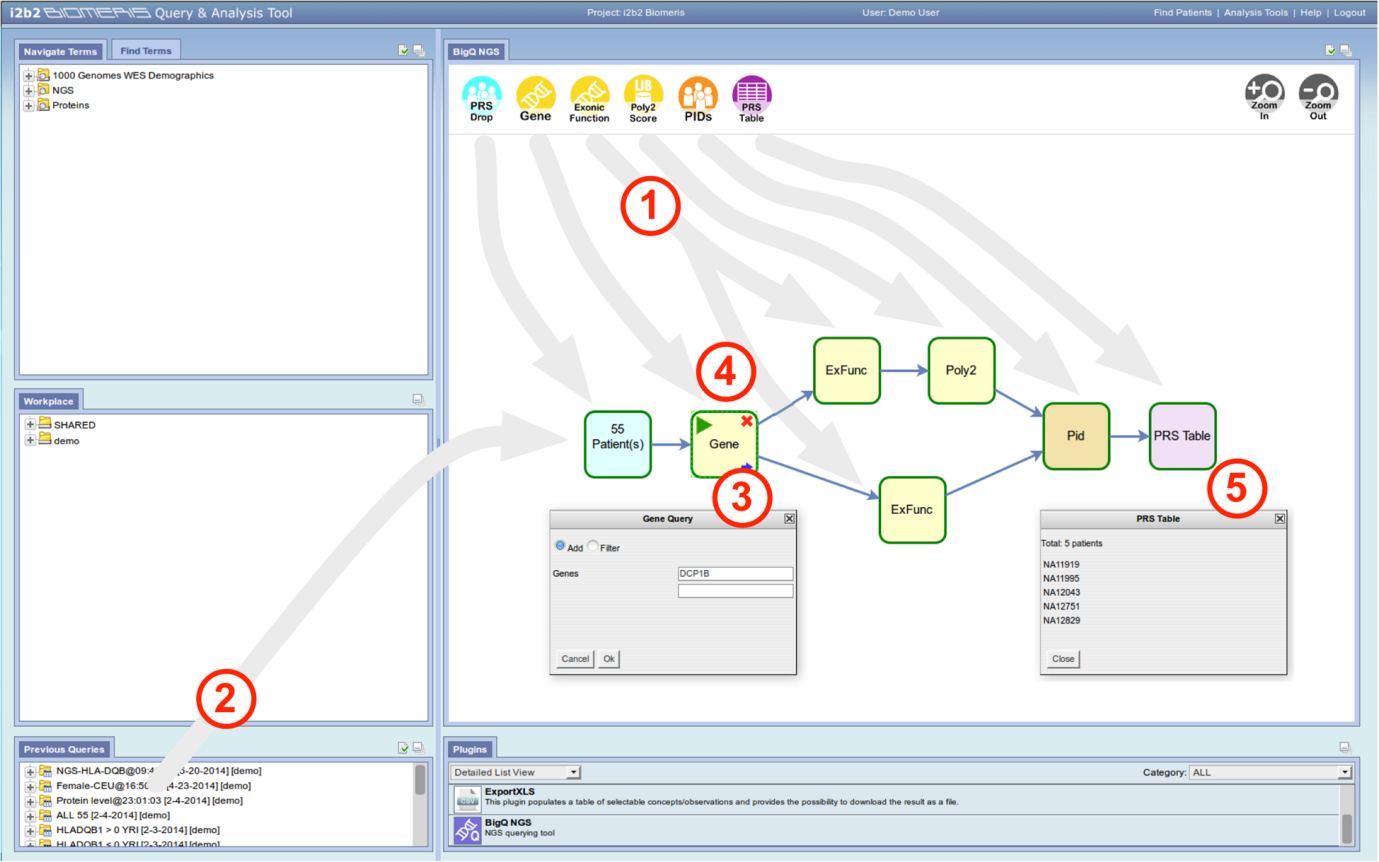
Screenshot of the BigQ-plugin with user interactions highlighted. (1) The user creates a query by dragging and dropping different blocks inside the plugin’s workspace, with each block representing a query on a single attribute that will be performed by the BigQ-cell. The query is defined by connecting the blocks to each other. (2) A patient set, previously created with a standard i2b2 query, is dragged and dropped on the Patient Result Set Drop (PRS Drop) block to define the patients whose exomes will be queried. (3) By double-clicking the standard query blocks (in yellow) it is possible to specify their query logic and query parameters. (4) The query process can start and each block executes its query sequentially, calling the BigQ-cell. (5) When all blocks have performed their query, the user can visualize the results by double-clicking the Patient Result Set Table (PRS Table) block

A typical interaction with the plugin starts with the user generating a patient set with the i2b2 Query Tool, using the data stored in the data warehouse; this allows extracting the patients corresponding to the phenotype of interest. Afterwards, moving to BigQ (in the plugins section) the user is presented with a blank workspace, where he/she can define the query as a sequence of blocks. Sequences typically start with the PRS Drop block that imports a patient set (with a drag-and-drop interaction) and makes it available to the blocks that are directly connected to it. Double-clicking on the query blocks brings up a form to specify their *query logic* and *query details*. Query blocks receive their *dataIn* from the upstream blocks, call the cell to run the query and return the *dataOut* generated by the cell to the downstream blocks. Finally the PRS Table block is added to show the result of the query. An example query is shown in Fig. 4.

## Results and discussion

To test our approach for integrating genetic queries within the i2b2 framework, we have performed a “stress test” on the system by submitting increasingly large Whole Exome Sequencing (WES) datasets of genomic variants. WES data were retrieved from the 1000 Genomes Project phase1 integrated release. We have tested our system on variant sets coming from 10, 20, 50, 100, 200 and 500 exomes. The average number of variants per exome, and thereby of the JSON documents added to the database for each individual, is about 23,000 for a maximum of 11,641,862 genomic variants in the case of 500 exomes.

**Fig. 5:**
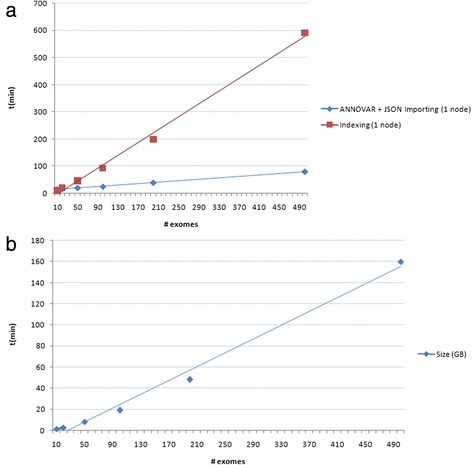
Importing time performances and disk space occupancy on a single machine. **a** Time performances for annotation, JSON conversion and importing of genomic variants belonging to 10,20,50,100,200,500 whole-exome samples into CouchDB, installed on a single Amazon AWS machine. **b** Disk space occupancy in relation to the whole-exome data growth

For the importing phase, BigQ-ETL has been tested on 8 Amazon EC2 [41] virtual machines, in particular, *c3.2xlarge* instances [42], a medium-high level server with 8 virtual CPUs and 15GB RAM. CouchDB was initially installed on a single *c3.2xlarge* instance. Total time to complete the ANNOVAR run, generating the JSON documents and uploading them for 500 exomes was approximately one hour and 20 min. The most computationally demanding operation in the data import process was indexing all the views in the database, with an average time of 1 min and 10 s per exome. Figure 5 reports disk space occupancy and importing time performances for annotation and indexing phase at the different dataset sizes.

For each data set we tested three types of queries that are commonly used by researchers to identify patients with possible variants of interest. The first query (Q1) searches among a limited patient set (5) those having a genomic variant with a given dbSNP id. The second query (Q2) is similar to Q1 with the difference that the search for the dbSNP id is performed on the whole patient dataset. The third query (Q3) aims at identifying patients that have variants either introducing a premature stop codon or non-synonymous variants with a high damaging score (according to PolyPhen-2) in a given gene and considering the whole patient dataset.

**Fig. 6:**
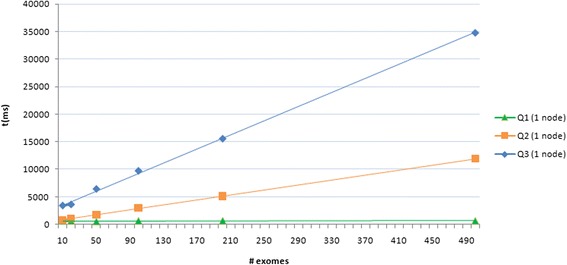
Query time performances. Query times (Q1, Q2 and Q3) plotted against the increasing numbers of individuals (i.e. variants) in the database

For each test we measured the average time necessary to run all three queries. Figure 6 and Table 1 show the results obtained, indicating that the query time is independent of the size of the database in the case of Q1, while it linearly scales with the size of the database in Q2 and Q3. It is interesting to note that with the proposed computational infrastructure the query time is almost instantaneous for the user in the case of Q1 (about 0.06 s), while querying more than 11 million variants (500 exomes) takes about 34 s. Since query flexibility is not provided by CouchDB, we have implemented a strategy to combine together the results from simple queries. As a consequence, a complex query (e.g. Q3) involving more than one variant attribute results in a longer query time due to the number of views to be searched (one for each attribute) and the *add* and/or *filter* operations to be performed in backend.

**Table 1:** Disk space usage and database queries response times for a growing number of exomes

# Exomes	Size (GB)	Q1 (ms)	Q2 (ms)	Q3 (ms)
10	1,1	669	825,6	3440,2
20	2,5	678,8	1065,4	3647,2
50	7,9	554,4	1745,2	6462
100	19	680,4	2956,2	9753,2
200	48	691	5129,4	15595,8
500	160	678,4	11897	34836,8

**Fig. 7:**
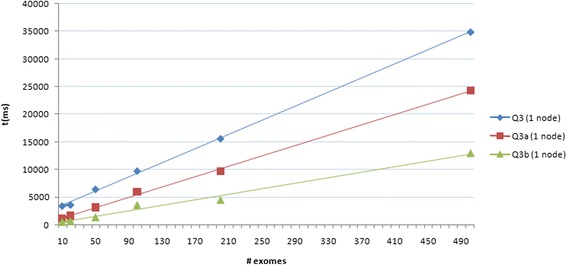
Query time performances using dedicated views. Time performance of the Q3 query and of those using dedicated views (Q3a and Q3b) plotted against the increasing numbers of individuals (i.e. variants) in the database

We therefore tried to perform query Q3 by building up two dedicated views, i.e. by creating JavaScript *map* functions that index variants basing on *patient id*, *gene*, *exonic function* and *PolyPhen-2 score*. In particular, the first view (Q3a) allows to dynamically set the gene name and the PolyPhen-2 threshold while for the second one (Q3b) the gene name and the PolyPhen-2 score are set a priori. As expected, query time decreased to about 24 and 13 s on 500 exomes for Q3a and Q3b respectively (see Fig. 7).

We also tested BigQ by using CouchDB in a distributed environment on the cloud. In particular, we have installed an elastic cluster version of CouchDB (BigCouch) on a 6 *c3.2xlarge* AWS machine. The distributed database was set up as follows: 6 shards (Q = 6), no redundant copies (*N* = 1), minimum read and write quorum (*R* = 1, *W* = 1); see Additional file 1 for details on BigCouch tuning parameters.

**Fig. 8:**
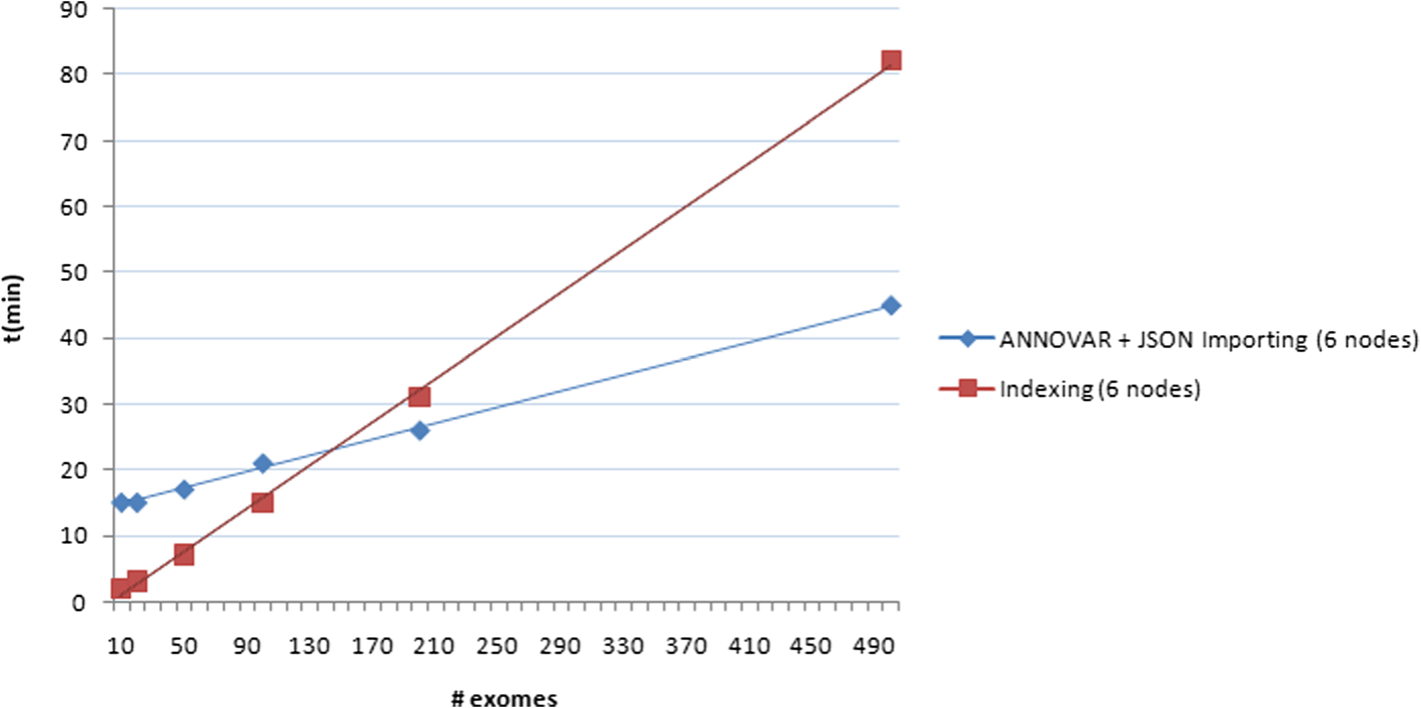
Importing time performances on a distributed environment. Time performance for annotation, JSON conversion and importing of genomic variants belonging to 10,20,50,100, 200, 500 whole-exome samples into CouchDB, installed on a distributed environment consisting of six Amazon AWS machines (*c3.2xlarge*)

We therefore performed the same operations described above, from data import to the query test. We found out that the computational time during import phase is reduced thanks to horizontal scaling: the view creation phase for the 500 exomes decreased from 9 h and 50 min (using a single node) to 1 h and 22 min (see Fig. 8).

**Fig. 9:**
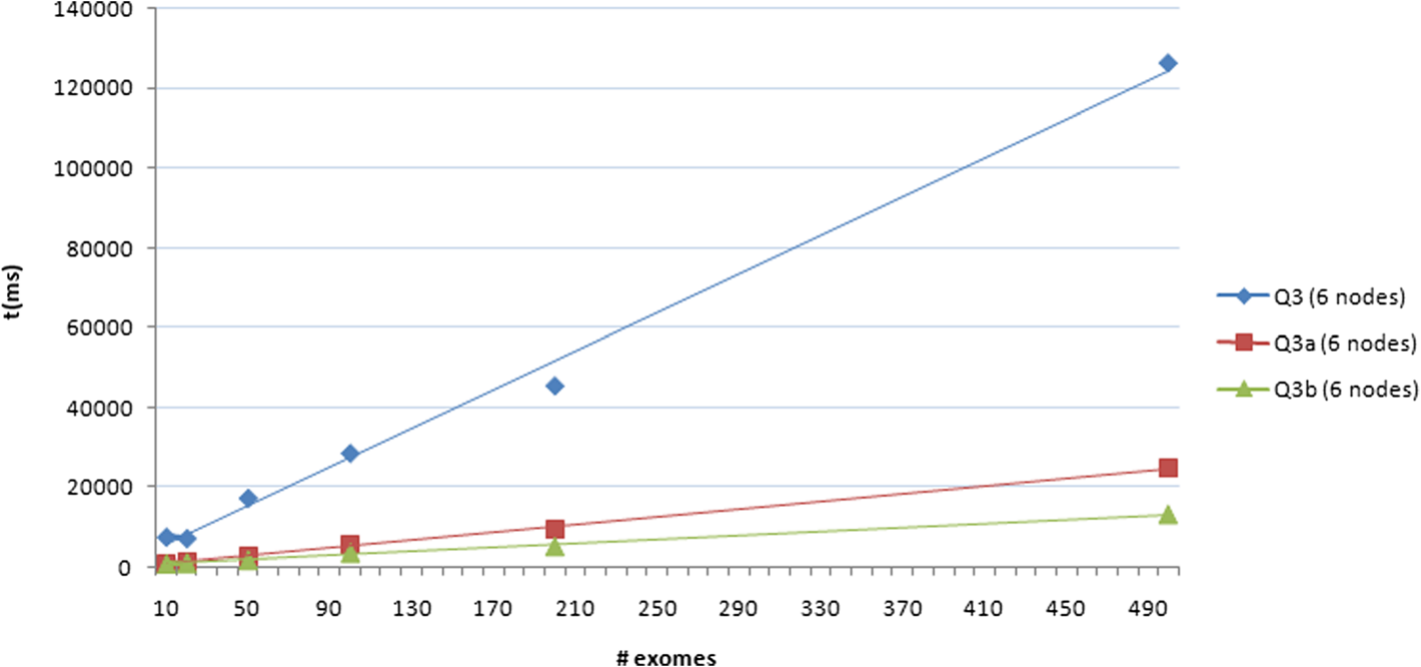
Query time performances on a distributed environment. Time performance of Q3, Q3a and Q3b using CouchDB in a distributed environment

Interestingly, we noted that horizontal scaling degrades performances on data retrieval (see Additional file 1: Figure S1), in particular for query Q3 if executed using the flexible schema that combines different queries, while no significant differences were observed when using the corresponding dedicated view (see Fig. 9).

This behavior can be explained by the BigCouch data sharding: each query needs to retrieve pieces of data of interest that are distributed among instances, to assemble them together and to return them to the client, resulting in slower performance especially when the number of sequential queries increases. As a consequence, the dedicated views strategy is even more necessary in a distributed CouchDB scenario. Therefore, we have planned to integrate BigQ-plugin with an additional functionality that allows to save a composed query by blocks, create ad hoc views and retrieve results in a faster way, improving system usability. Because each research group tends to standardize the way it searches for variants of interest, we believe this approach is valuable.

Furthermore we intend to explore the fine-tuning features available for BigCouch. These tunings, together with an optimal number of nodes in the cluster, could bring about a considerable improvement in the indexing/query performance of the system.

## Conclusions

In this paper we have described a system designed to deal with many genomic variants coming from heterogeneous and frequent NGS analysis, performed in a hospital environment where clinical research data are managed by the i2b2 framework.

The integration of patient clinical phenotype and genomic variant profiles is a two-step process: first patient cohorts are generated by querying clinical terms using the i2b2 built-in functionalities and second, the cohort is uploaded to the BigQ-plugin in order to retrieve the corresponding genomic variants of interest.

Variants are annotated with useful biological data by ANNOVAR software and stored in the document-based NoSQL CouchDB system. The data are then managed by a dedicated i2b2 cell and a visual-programming plugin for easily performing queries.

The system has been conceived also to deal with variants of unknown clinical meaning and generated by different NGS applications, possibly characterized by useful but heterogeneous biological data. For this reason, the data model is flexible, and adherent to the contents of ANNOVAR documents; the database can thus be easily updated with new versions of the variant annotations.

The query system has very promising performance, showing to scale well with the database volume, making it feasible to jointly query clinical and genetic data. We note that the choice of CouchDB allows naturally relying on cloud-based implementations on elastic clusters, such as the BigCouch system. Despite i2b2 instances are usually installed locally (relying on the hospital hardware infrastructure) and not provided as a Software as a Service module, one could in fact use Amazon AWS products to build its own i2b2 infrastructure on the cloud.

In the future we will compare our implementations to other state of the art extensions of i2b2 and TRANSMART developed to deal with NGS data, and we will work on other plugins, in order to better enable the full exploitation of NGS data within the i2b2 infrastructure.

## Availability and requirements

- **Project name:** BigQ.
- **Project home page:**http://www.biomeris.com/index.php/en/tasks/bigq-ngs-en.
- **Operating system(s):** Linux.
- **Programming language:** Java, Perl.
- **License:** GNU General Public License.

## Additional file

Additional file 1:
**This file contains supplementary tables, figures and BigCouch tuning parameters.**(DOCX 43 kb)

## Abbreviations

i2b2: informatics for integrating biology and beside
NGS: next generation sequencing
NoSQL: not only SQL
ETL: extraction, transformation and loading
1KGP: 1000 genomes project
VCF: variant calling format
