# Supplementary material for: BigQ: a NoSQL based framework to handle genomic variants in i2b2
Source: BMC Bioinformatics. 2015 Dec 29;16:415. doi: 10.1186/s12859-015-0861-0 (PMC4696314; doi:10.1186/s12859-015-0861-0)
Supplement: Additional file 1: — This file contains supplementary tables, figures and BigCouch tuning parameters.(DOCX 43 kb) [file 12859_2015_861_MOESM1_ESM.docx]

# Supplementary Material

**BigCouch tuning parameters**

In addition to the number of nodes, BigCouch databases are governed by 4 parameters: the number of shards (Q), the number of redundant copies of each shard (N), the read quorum (R) and the write quorum (W). The number of shards represents the number of partitions forming the database; documents are deterministically mapped to a specific shard on the base of their id; Q is an integer greater than 0. The number of redundant copies of each shard specifies how many times each shard has to be replicated along the database; N is an integer greater than 0.

While the number of nodes is set up when the BigCouch cluster is created, Q and N must be provided whenever a new database is created inside the datastore.

Finally the quorum parameters R and W (both less than or equal to N) are specified at query time and represent the number of identical document copies that must be respectively read and saved before a reading or writing operation can be considered successful. R and W can be specified at query time.

FIGURES

**Figure 1.** Query times (Q1, Q2 and Q3) plotted against the increasing dataset size and using CouchDB in a distributed environment with 6 Amazon AWS machines (*c3.2xlarge*).

**
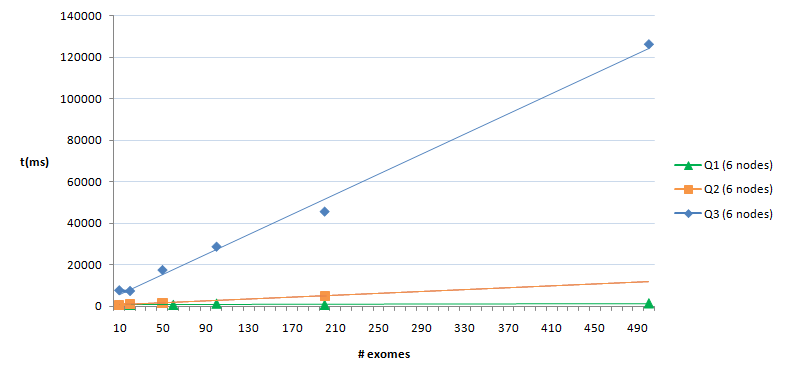
**

TABLES

**Table 1.** Variant annotation attributes stored within each JSON document.

| **Field Name** | **Type** | **Description** |
| --- | --- | --- |
| chr | String | Chromosome |
| ref | String | Reference |
| obs | String | Variant |
| function | String | Variant function |
| refGenome | String | Genome assembly |
| start | Integer | Mutation start position |
| end | Integer | Mutation end position |
| gene name | String | Gene symbol (refSeq) |
| segDup | Double | Sequence identity score for the segmental duplication region where variant is located in |
| AVSIFT | Double | Whole-exome SIFT scores for non-synonymous variants |
| exonicFunc | String | Exonic variant function |
| exonic_hgvs_transcript | String | Variant in hgvs format on transcript |
| exonic_hgvs_protein | String | Variant in hgvs format on protein |
| exonic_exon | Integer | Exon number where variant is located in |
| gt | String | Genotype |
| vcf line | String | Original VCF line |
| 1kgp_freq | Double | Variant frequency for 1KGP |
| 1kgp_version | String | 1KGP version |
| dbSNP_id | String | dbSNP identification id |
| dbSNP_version | String | dbSNP version |
| dbESP_freq | Double | Variant frequency for ESP |
| dbESP_version | String | ESP version |
| LJB_phyloP_score | Double | Evolutionary conservational score by phyloP |
| LJB_SIFT_score | Double | SIFT scores for non-synonymous variants |
| LJB_PolyPhen-2_HDIV_score | Double | PolyPhen-2 scores for non-synonymous variants (hdiv model) |
| LJB_PolyPhen-2_HDIV_pred | String | PolyPhen-2 class for non-synonymous variants (hdiv model) |
| LJB_mutationTaster_score | Double | MutationTaster scores for non-synonymous variants |
| LJB_mutationTaster_pred | String | MutationTaster class for non-synonymous variants |
| LJB_GERP | Double | Evolutionary conservational score by GERP |
| LJB_PolyPhen-2_HVAR_score | Double | PolyPhen-2 scores for non-synonymous variants (hvar model) |
| LJB_PolyPhen-2_HVAR_pred | String | PolyPhen-2 class for non-synonymous variants (hvar model) |
| LJB_mutationAssessor_score | Double | MutationAssessor scores for non-synonymous variants |
| LJB_mutationAssessor_pred | String | MutationAssessor class for non-synonymous variants |
| LJB_fathm_score | Double | FATHM scores for non-synonymous variants |
| LJB_siPhy_score | Double | Evolutionary conservational score by Siphy |
| LJB_ LRT_score | Double | LRT scores for non-synonymous variants |
| LJB_LRT_pred | String | LRT class for non-synonymous variants |
| LJB_version | String | dbNSFP version |
